# Stereotactic body radiotherapy for localized prostate cancer: disease control and quality of life at 6 years

**DOI:** 10.1186/1748-717X-8-118

**Published:** 2013-05-13

**Authors:** Alan J Katz, Michael Santoro, Fred Diblasio, Richard Ashley

**Affiliations:** 1 Flushing Radiation Oncology, 40-20 Main St, Flushing, NY 11354, USA; 2 Mount Sinai School of Medicine, New York, NY, USA; 3 North Shore LIJ System, Manhasset, NY, USA

**Keywords:** Prostate, Stereotactic body radiotherapy, CyberKnife

## Abstract

**Background:**

Stereotactic body radiotherapy (SBRT) may yield disease control for prostate cancer in a brief, hypofractionated treatment regimen without increasing treatment toxicity. Our report presents a 6-year update from 304 low- (n = 211), intermediate- (n = 81), and high-risk (n = 12) prostate cancer patients who received CyberKnife SBRT.

**Methods:**

The median PSA at presentation was 5.8 ng/ml. Fifty-seven patients received neoadjuvant hormonal therapy for up to one year. The first 50 patients received a total dose of 35 Gy in 5 fractions of 7 Gy. The subsequent 254 patients received a total dose of 36.25 Gy in 5 fractions of 7.25 Gy. Toxicity was assessed with the Expanded Prostate Cancer Index Composite questionnaire and the Radiation Therapy Oncology Group urinary and rectal toxicity scale. Biochemical failure was assessed using the nadir + 2 definition.

**Results:**

No patients experienced Grade III or IV acute complications. Fewer than 5% of patients experienced any acute Grade II urinary or rectal toxicities. Late urinary Grade II complications were observed in 4% of patients treated to 35 Gy and 9% of patients treated to 36.25 Gy. Five (2%) late Grade III urinary toxicities occurred in patients who were treated with 36.25 Gy. Late Grade II rectal complications were observed in 2% of patients treated to 35 Gy and 5% of patients treated to 36.25 Gy. Bowel and urinary quality of life (QOL) scores initially decreased, but later returned to baseline values. An overall decrease of 20% in the sexual QOL score was observed. QOL in each domain was not differentially affected by dose. For patients that were potent prior to treatment, 75% stated that they remained sexually potent. Actuarial 5-year biochemical recurrence-free survival was 97% for low-risk, 90.7% for intermediate-risk, and 74.1% for high-risk patients. PSA fell to a median of 0.12 ng/ml at 5 years; dose did not influence median PSA levels.

**Conclusions:**

In this large series with long-term follow-up, we found excellent biochemical control rates and low and acceptable toxicity, outcomes consistent with those reported for from high dose rate brachytherapy (HDR BT). Provided that measures are taken to account for prostate motion, SBRT’s distinct advantages over HDR BT include its noninvasiveness and delivery to patients without anesthesia or hospitalization.

## Introduction

Conventional treatments for localized prostate cancer target local control at the potential expense of morbidity and decreased quality of life. Urinary function impairment occurs in 5-28% of patients at 2 years after radical prostatectomy (RP) and in 2-14% of patients at 2 years after external-beam radiation therapy (EBRT) [1,2]. Bowel distress is found in 3-21% of RP patients and 8-37% of EBRT patients 2 years after treatment [1]. Erectile dysfunction has been reported in 51-82% and 30-51% of patients 2 years after RP and EBRT, respectively [1,3,4]. Sexual quality-of-life (QOL) estimates show similar results for these treatments [2], although it should be noted that the radiotherapy patients in this study were older on average, and therefore more likely to have lower sexual QOL. Indeed, the rate of such complications and the extent to which they reduce the QOL of prostate cancer patients contributed to a recent recommendation from the United States Preventive Services Task Force (USPTF) against routine prostate-specific antigen (PSA) screening for prostate cancer in men age 75 or older [5]. Furthermore, the rate of complications and decreased QOL has prompted researchers to consider using stereotactic body radiotherapy (SBRT) (i.e., highly targeted radiotherapy with large daily doses of radiation) to try to increase disease control while decreasing side effects.

Radiobiologically, slowly proliferating prostate cancer cells are thought to have a low α/β ratio; in two recent reviews of studies in which the fractional dose was varied, the α/β ratio continued to average about 1.5 Gy [6,7], consistent with the earliest estimates of Brenner and Hall [8]. This low α/β ratio suggests that prostate cancer cells have a high sensitivity to dose per fraction. This sensitivity suggests that a hypofractionated radiation delivery regime with a large radiation dose delivered in a smaller number of fractions may be advantageous.

The first reported hypofractionated radiation therapy treatments for prostate cancer occurred in the early 1960s [9]. Treatments of 36 Gy delivered in 6 equal fractions were motivated by resource limitations rather than radiobiology. Nevertheless, two decades of follow-up has confirmed that this regimen leads to favorable and long-term local response, survival, and safety. Subsequently, hypofractionated prostate cancer treatment has been performed with EBRT in per-fraction doses of 2.5-3.1 Gy [10-13], with brachytherapy (BT) in per-fraction doses of 5.5-11.5 Gy [14-16], and with linac-based SBRT in per-fraction doses of 6.7 Gy in 5 fractions [17]. In the first paper to report on CyberKnife® SBRT (Accuray Incorporated, Sunnyvale, CA), King et al. reported a median 33-month follow-up for patients that received 5 fractions of 7.25 Gy (for a total dose of 36.25 Gy). They did not observe any biochemical failure and the early and late toxicity profiles of their patients were no worse than equivalent historical cohorts treated with conventional EBRT [18]. We found similar results in an earlier paper that discussed 304 patients who were treated with CyberKnife and had a limited median follow-up of 19 months [19]. At 19 months, toxicity was low and early PSA control was encouraging. Other reports have since been published that found similarly low toxicity and high efficacy [20-24]. In a study of 41 low-risk patients with the longest follow-up from the combined Stanford and Naples, Florida groups, Freeman and King [25] reported a 5-year biochemical disease-free survival rate of 93% that was accompanied by low toxicity. Thus, although long-term follow-up is limited, hypofractionated treatment of prostate cancer can result in effective biochemical control while maintaining low rectal and bladder toxicities.

Our report presents a 6-year update of treatment results from 304 low-, intermediate-, and high-risk prostate cancer patients who received CyberKnife SBRT. Particular attention is given to biochemical control and urinary, rectal, and sexual toxicities.

## Methods and materials

### Patient population

Data were analyzed for all clinically localized prostate cancer patients who were treated with CyberKnife SBRT at Winthrop University between April 2006 and July 2008. The treatment protocol was IRB-approved and the first 15 patients were treated in a prospective fashion to assess the feasibility of the approach in our hands. Subsequent patients were treated according to this approved protocol, but not as part of a prospective study. All patients provided informed consent for their outcomes to be incorporated in this retrospective study. All 304 patients had adenocarcinoma of the prostate. Of these patients, 280 (92.2%) of them presented with clinical stage T1c N0 M0 and 24 (7.8%) presented with clinical stage T2a N0 M0 (as determined by a physical exam and bone and CT scans). The median PSA at presentation was 5.8 ng/ml (range, 0.7-27.3 ng/ml). Table 1 details the patient characteristics. All patients signed consent statements and were informed of the potential risks involved with CyberKnife treatment. The treatment protocol received institutional review board approval.

**Table 1:** Patient characteristics at diagnosis

**Age at diagnosis**	**Years**	
Mean (range)	69.2 (45 – 88)	
**Age at diagnosis**	**Number of Patients**	**Percent of Patients**
45–49	1	0.3
50–54	7	2.3
55–59	23	7.6
60–64	35	11.5
65–70	54	17.8
70–74	80	26.3
75–79	54	17.8
80–84	36	11.8
85-88	14	4.6
**PSA level at diagnosis**	**ng/mL**	
Mean (range)	6.08 (0.7 to 27.7)	
Median	5.8	
**PSA level at diagnosis**	**Number of Patients**	**Percent of Patients**
<4 ng/mL	59	19.4
4–10 ng/mL	203	66.8
>10–20 ng/mL	40	13.2
>20 ng/mL	2	0.7
**Clinical Stage**	**Number of Patients**	**Percent of Patients**
T1cN0M0	280	92.1
T2aN0M0	24	7.9
**Gleason Score**	**Number of Patients**	**Percent of Patients**
= 6	222	73
=7	70	23
> 8	12	4
**Hormone Treatment**	**Number of Patients**	**Percent of Patients**
No	247	81.3
Yes	57	18.8
**Risk Assessment: Criteria**	**Number of Patients**	**Percent of Patients**
Low Risk: Gleason Score ≤ 6 *and* PSA ≤ 10 ng/ml.	211	69.4
Intermediate Risk: Gleason = 7 *or* PSA > 10 *and* PSA < 20	81	26.6
High Risk: Gleason ≥ 8 *or* PSA > 20	12	0.7

### Hormone therapy

Fifty-seven patients received neoadjuvant hormonal therapy. As this therapy was usually stopped at the time of consultation, 29 (51%) of those patients received it for up to three months. The remaining 28 patients (49%) received hormone therapy for up to one year at the discretion of the patient’s urologist.

### Treatment planning and delivery

Image-guided SBRT was delivered to all patients using the CyberKnife with Multiplan® inverse treatment planning and motion tracking throughout treatment based on internal fiducials. A detailed description of the CyberKnife system can be found elsewhere [26].

Approximately 2 weeks before treatment planning, 4 gold fiducial seeds were placed transperineally in each patient to allow for motion tracking during treatment. Two of the seeds were implanted at the prostate apex and two were implanted at its base. After allowing time for possible seed migration, treatment planning was performed prior to the treatment day using a CT scan (1.5-mm cuts) with MRI fusion. All pretreatment imaging was performed with the patient in the same position that was used for his treatment delivery. For low-risk patients, just the prostate made up the gross target volume (GTV). For intermediate- to high-risk patients who had a Gleason Score of greater than 6 and a PSA of greater than 15 ng/ml, the proximal half of the seminal vesicles was added to the GTV. After the GTV was delineated, a margin was added to create the planning target volume (PTV). For low- and intermediate-risk patients, the margin was extended 5 mm on all sides except for posteriorly (by the rectum) where a 3-mm margin was used. For high-risk patients, an 8-mm margin was added to the involved side. All patients had the bladder, prostate, rectum, seminal vesicles, and penile bulb contoured; the urethra was not identified.

SBRT was delivered at two dose levels. The first 50 patients (16%) received a total dose of 35 Gy in 5 fractions of 7 Gy each to cover at least 96% of the PTV. The subsequent 254 patients (84%) received a total dose of 36.25 Gy in 5 fractions of 7.25 Gy to cover at least 96% of the PTV. The dose was increased to 7.25 Gy per fraction when preliminary reports at scientific meetings indicated that the higher dose could be delivered safely (based on early results of the study by King et al. of Stanford University; [18]). The mean number of beams was 152 (range, 140–170). The mean D50 to the bladder and rectum was 43% and 41% of the prescribed dose, respectively.

Treatments were performed on five consecutive days. In the morning before each treatment, patients completed a bowel prep that included Dulcolax® (Boehringer Ingelheim, Ingelheim, Germany) and a Fleet enema (C.B. Fleet Company, Inc., Lynchburg, Virginia). In addition, at least 15–20 minutes before treatment all patients received 1500 mg of amifostine (MedImmune, LLC, Gaithersburg, MD) that was mixed in saline and instilled into the rectum.

### Follow-up schedule and toxicity assessment

Each patient was seen for follow-up three weeks after his final treatment, four months after that, and every six months thereafter. After two years, follow-up was done annually. Toxicity was assessed at every follow-up visit and used the Expanded Prostate Cancer Index Composite (EPIC) questionnaire [27] and the Radiation Therapy Oncology Group (RTOG) urinary and rectal toxicity scale. Acute toxicity was defined as those events that presented and resolved within the first 3 months following treatment. PSA was assessed by the referring urologist 3 and 6 months after treatment and every 6 months thereafter. Biochemical failure was the end point of the study and used the Phoenix (nadir + 2) biochemical failure definition [28].

## Results

### Follow-up

The median follow-up for all patients was 60 months (range, 8–78 months). Patients who received the higher dose (36.25 Gy) had a median follow-up of 60 months (range, 8–72 months). For the lower dose (35 Gy), the median follow-up was 72 months (range, 9–78 months). Sixteen patients were lost to follow-up. Although there were 5 deaths in the 35 Gy group and 21 deaths in the 36.25 Gy group, none of these deaths were due to prostate cancer.

### Acute toxicity

Except for one patient who died from causes other than prostate cancer at 4 months, all patients received a toxicity follow-up at 3 weeks and 5 months. Acute toxicity profiles were thus collected for 303 patients. Table 2 presents the RTOG-scale-graded acute urinary and rectal toxicities that were observed during the first 3 months as a function of treatment dose. No patients experienced any Grade III or IV acute complications. Fewer than 5% of patients (14/303) experienced any acute Grade II urinary or rectal toxicities.

**Table 2:** Acute bladder/rectal toxicity using RTOG scoring after prostate treatment using the 35 and 36.25 Gy doses

		**RTOG grade% (number) of patients**			
	**Total dose**	**0**	**I**	**II**	**III & IV**
**Acute urinary**	35 Gy	24% (12)	72% (36)	4% (2)	–
	36.25 Gy	20.5% (52)	74.8% (190)	4.7% (12)	–
**Acute rectal**	35.00 Gy	20% (10)	76% (38)	4% (2)	–
	36.25 Gy	22.% (56)	74.4% (189)	3.5% (9)	–

### Late toxicity

**Figure 1:**
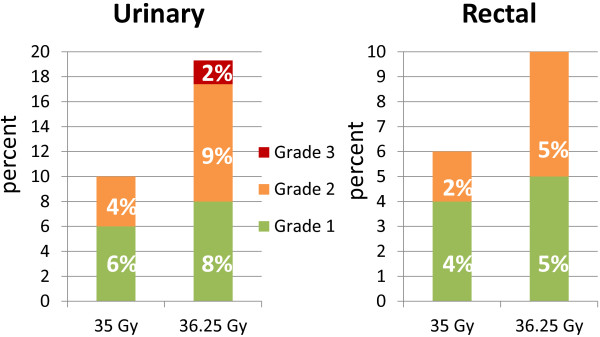
RTOG-graded late toxicity for patients treated with 35 or 36.25 Gy.

Figure 1 presents late urinary and rectal toxicities and differentiates them for all patients by dose. Late urinary Grade II complications were observed in 4% of patients treated to 35 Gy and 9% of patients treated to 36.25 Gy. Five (2%) late Grade III urinary toxicities occurred in patients who were treated with 36.25 Gy. Although a difference in late urinary complication rates was observed between patients who received 35 Gy or 36.25 Gy, this observation was not statistically significant (*p* > 0.5). Late rectal Grade II complications were observed in 2% of patients treated to 35 Gy and 5% of patients treated to 36.25 Gy. Late rectal complications also did not differ between groups (*p* > 0.5).

### Quality of life

**Figure 2:**
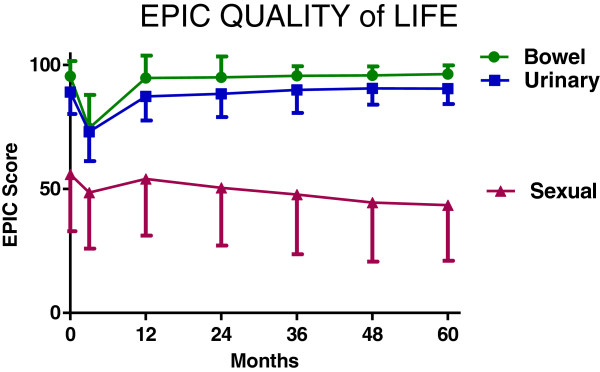
**Mean EPIC quality of life scores.** Under the figure are percentages of patients reaching each time point that completed the EPIC ((number completing EPIC / number at risk) X 100).

**Figure 3:**
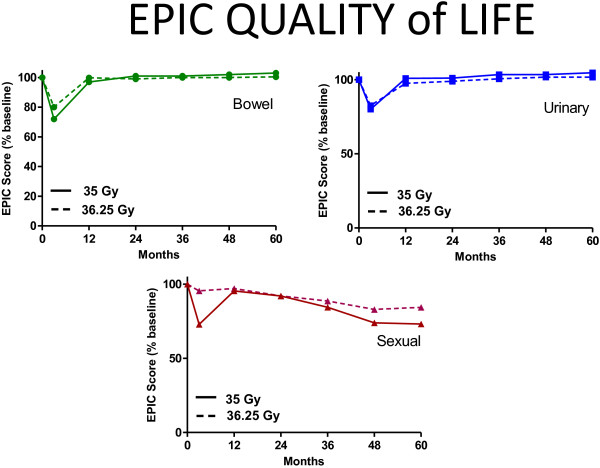
**Mean EPIC quality of life scores as a function of dose.** All differences were not significant (p > 0.05).

All patients completed the initial EPIC questionnaire prior to treatment. For subsequent time points, the number of patients that completed this questionnaire varied, depending on how many patients reached each follow-up time point and also completed the questionnaire. Figure 2 shows the EPIC scores for bowel, urinary, and sexual QOL. Bowel and urinary QOL scores initially decreased, but then returned to baseline values. For sexual QOL, an overall gradual decrease of about 10% in the QOL score was observed. QOL in each domain was not differentially affected by dose (see Figure 3). To further examine sexual QOL and determine if patients remained potent, we verbally screened patients that were potent prior to treatment (n = 228). At a median 60 months follow-up (range, 48–78 months), 75% percent of them (172/228) stated that they remained sexually potent; 25% of these patients required medication. EPIC QOL scores are presented as a function of dose in Figure 3. In no case was dose a significant determinant of QOL (*p* < 0.05).

### Biochemical control and PSA

**Figure 4:**
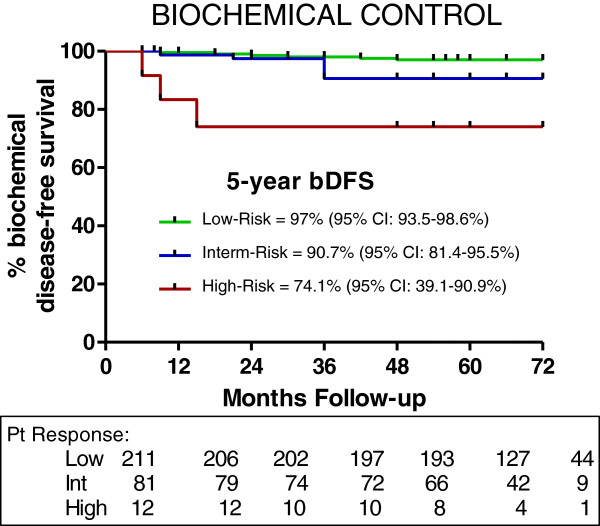
**Kaplan-Meier biochemical disease-free survival for each risk group**.

**Figure 5:**
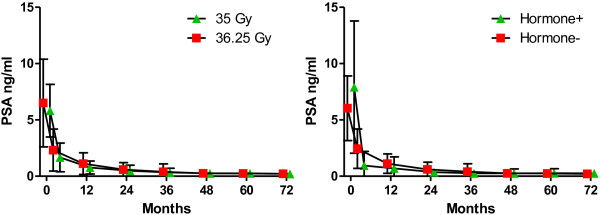
**Median PSA at baseline and after SBRT for both dose groups and for patients treated with or without hormones (=/- standard deviation).** Dose did not significantly alter PSA levels over time.

Actuarial 5-year biochemical recurrence-free survival was 97% for low-risk, 90.7% for intermediate-risk, and 74.1% for high-risk patients (Figure 4). For low-risk patients, there was no difference in biochemical disease-free survival (BDFS) as a function of dose, ie 35 Gy vs 36.25 Gy (98% vs 97%). In fact, 43 low-risk and 7 intermediate-risk patients that were treated with 35 Gy had a BDFS of 98% at 6 years. In the intermediate-risk category, patients with a Gleason score of 4 + 3 had a 5-year BDFS of 84% vs a bRFS of 95% for those with a Gleason score of less than 4 + 3. PSA fell to a median of 0.12 ng/ml at 5 years; dose did not influence median PSA levels (see Figure 5). PSA for hormone-treated patients versus those not treated with hormones is also shown in Figure 5. PSA was clearly lower at the 3-month time point for hormone-treated patients, but not at other time points. A PSA bounce of greater than 0.2 ng/ml occurred in 51/304 (17%) of patients with a median time-to-bounce of 30 months. The median bounce was 0.55 ng/ml.

## Discussion

In this large series with long-term follow-up, we found excellent biochemical control rates and low and acceptable toxicity. PSA fell steadily after treatment and achieved very low levels (mean of 0.25 ng/ml) within 4–5 years, findings that portend good long-term disease control outcomes [29,30]. These findings support an estimate of the α/β ratio of 1.5 Gy. A ratio of 1.5 Gy means that we have delivered an equivalent dose (EQD) of 90–96 Gy at 1.8 Gy per fraction, an EQD which accounts for the higher control rates than those seen with the use of 81 Gy [29]. Although our results with high-risk patients are encouraging, it is important to note that our study included only 12 patients and more data is necessary to confirm these findings.

Our outcomes are consistent with those that have resulted from high dose rate brachytherapy (HDR BT), with or without EBRT [15,16]. In a recent paper Demanes et al. [14] reported an 8-year recurrence-free survival of 97% in a mixed cohort of low and intermediate-risk patients. If additional follow-up confirms that this level of long-term disease control can be obtained with SBRT, SBRT’s advantages over HDR BT, primarily its non-invasiveness and ability to deliver treatment to patients without anesthesia or hospitalization may make it the preferred modality.

We employed two dose levels in our study. We initially treated patients with 35 Gy but escalated to 36.25 Gy six months into the study after observing low acute toxicity at 35 Gy and after reports from others of acceptable toxicity at a dose of 36.25 Gy. Based on current data, however, the higher dose does not appear to be necessary for low and low-intermediate patients. No difference in PSA control or nadirs were seen between the two doses, a finding which corroborated a recent matched-pairs study with 48 month follow-up [31]. A trend to increased toxicity with the 36.25 Gy dose was observed. It is possible that these events did not rise to the level of statistical significance due to the small number of patients within the 35 Gy group. Due to these findings, we resumed treating low- and low-intermediate risk patients with 35 Gy soon after the present study was completed. With more patients and longer follow-up a significant improvement in toxicity at the lower dose may be observed, in which case 35 Gy may be the optimal dose to assure long-term disease control and low toxicity. Such a finding would imply a flattening of the biologically equivalent dose response curve from 90–96 Gy EQD (assuming an α/β ratio of 1.5 Gy).

Our results are supported by a recent study of 1101 patients in a pooled analysis from eight institutions [32], reported at the 2012 meeting of the American Society of Radiation Oncology (ASTRO). This analysis reported only on biochemical control outcomes and found 96%, 92%, and 80% control with five-year actuarial follow-up for low-, intermediate- and high-risk patients, respectively. These results excluded the PSA failures that subsequently resolved on their own (i.e., “bounces”). Importantly, the three-year median follow-up results were excellent (at 80% control) for more than 100 patients with high-risk disease. These outcomes approximate those obtained in the current study, as well as from a study by Katz et al. [33] that reported long-term follow-up results for high-risk patients that received a CyberKnife boost after EBRT. In this study a biochemical control rate of 77.7% at 3 years was obtained for high-risk patients who received 45 Gy to the pelvis followed by a CyberKnife boost of 18–21 Gy. No differences were found between patients who received 35 Gy or doses as high as 40 Gy. The use of ADT also did not affect outcomes. Longer follow-up with more patients is warranted before firm conclusions can be made about the efficacy of SBRT monotherapy or SBRT as a boost for these patients at a higher risk for disease outside the prostate.

Because surgery is often used instead of radiation to treat prostate cancer, patients need information on both disease control and QOL changes associated with either modality. To better gauge the impact of prostate cancer treatment on QOL, one study compared the QOL responses from a large group of patients who had recently received CyberKnife SBRT to those of a similar group who instead underwent open surgery [34]. EPIC scores were used to assess QOL. For all time intervals up to 36 months, the patients who received SBRT had superior EPIC scores (in terms of urinary and sexual domains) than those who underwent surgery. Bowel domain was slightly worse in the short term for those in the SBRT group, but patients in both the SBRT and surgery groups had excellent preservation of bowel function after 12 months. It is important to note that surgical patients underwent open prostatectomy. It is possible that improvements in prostatectomies, including the use of laparoscopic techniques, will improve QOL post-surgery. On the other hand, SBRT patients in this study were older, on average, a factor that could have swayed QOL in favor of surgery. Although it is clearly not appropriate to assert forcefully the superiority of SBRT over surgery based on the data in this study, there is at least no evidence that SBRT results in *poorer* QOL outcomes for prostate cancer patients.

## Conclusions

In this study of 304 patients followed out to 6 years, we found excellent biochemical control rates with low and acceptable toxicity. Provided prostate motion is tracked and accounted for, high-dose, hypofractionated SBRT for prostate cancer appears that it may be an attractive treatment option for patients with low- and intermediate-risk disease. Longer-term follow-up with additional patients is needed to firmly assess efficacy and toxicity of SBRT relative to other, more established approaches, and its utility in high-risk patients.

## Competing interests

Dr. Katz has received speaker’s honoraria from Accuray, Inc., Sunnyvale CA. The remaining authors declare that they have no competing interests.

## Authors’ contributions

AK was responsible for the treatment of the patients, collection of data, interpretation of data and manuscript preparation. MS, FD, and RA were responsible for gathering and interpreting data, manuscript revision and final manuscript approval. All authors read and approved the final manuscript.
